# Revolutionizing the construction industry by cutting edge artificial intelligence approaches: a review

**DOI:** 10.3389/frai.2024.1474932

**Published:** 2024-12-12

**Authors:** Eliezer Zahid Gill, Daniela Cardone, Alessia Amelio

**Affiliations:** ^1^Department of Engineering and Geology, University “G. d’Annunzio” Chieti-Pescara, Pescara, Italy; ^2^HPC Laboratory, Department of Engineering and Geology, University “G. d’Annunzio” Chieti-Pescara, Pescara, Italy

**Keywords:** constructions 4.0, machine learning, air pollutants, concrete, physiological signals, environmental sustainability, predictive analysis, intelligent monitoring

## Abstract

The construction industry is rapidly adopting Industry 4.0 technologies, creating new opportunities to address persistent environmental and operational challenges. This review focuses on how Artificial Intelligence (AI), Machine Learning (ML), and Deep Learning (DL) are being leveraged to tackle these issues. It specifically explores AI’s role in predicting air pollution, improving material quality, monitoring worker health and safety, and enhancing Cyber-Physical Systems (CPS) for construction. This study evaluates various AI and ML models, including Artificial Neural Networks (ANNs) and Support Vector Machines SVMs, as well as optimization techniques like whale and moth flame optimization. These tools are assessed for their ability to predict air pollutant levels, improve concrete quality, and monitor worker safety in real time. Research papers were also reviewed to understand AI’s application in predicting the compressive strength of materials like cement mortar, fly ash, and stabilized clay soil. The performance of these models is measured using metrics such as coefficient of determination (*R*^2^), Root Mean Squared Error (RMSE) and Mean Absolute Error (MAE). Furthermore, AI has shown promise in predicting and reducing emissions of air pollutants such as PM2.5, PM10, NO_2_, CO, SO_2_, and O_3_. In addition, it improves construction material quality and ensures worker safety by monitoring health indicators like standing postures, electrocardiogram, and galvanic skin response. It is also concluded that AI technologies, including Explainable AI and Petri Nets, are also making advancements in CPS for the construction industry. The models’ performance metrics indicate they are well-suited for real-time construction operations. The study highlights the adaptability and effectiveness of these technologies in meeting current and future construction needs. However, gaps remain in certain areas of research, such as broader AI integration across diverse construction environments and the need for further validation of models in real-world applications. Finally, this research underscores the potential of AI and ML to revolutionize the construction industry by promoting sustainable practices, improving operational efficiency, and addressing safety concerns. It also provides a roadmap for future research, offering valuable insights for industry stakeholders interested in adopting AI technologies.

## 1 Introduction

**Figure 1:**
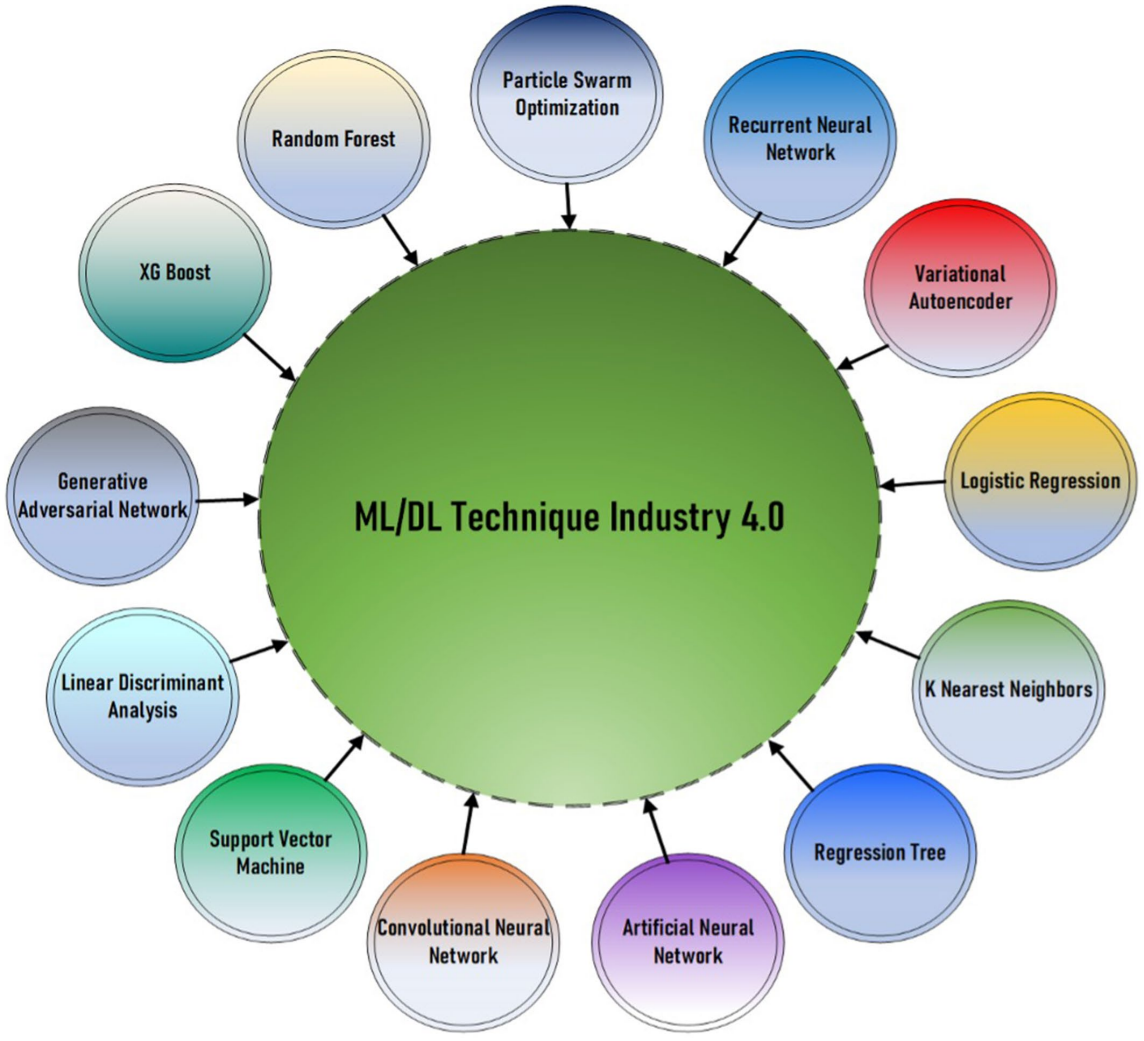
Mind mapping illustration of the ML/DL techniques applied in the construction sector (Al-Janabi et al., 2019).

Combining the technologies of AI and ML with construction techniques has a lot of potential in solving long-standing problems. Furthermore, the AI and ML literature in construction expands beyond individual examples to create a structure of methods, data, and results. Thus, every research endeavor proves the scalability and versatility of AI and ML algorithms in handling construction-related issues in various fields. In the literature, different papers describe the expedition of assessment models as the cutting-edge sole solution for the construction industry where the approximation of air pollutant concentrations in the atmospheric environment, concrete performance, and workforce safety are identified. It is a prerequisite to display the distribution of the AI techniques concerning Industry 4.0 (see Figure 1). The present work introduces a more extensive research field in which AI and ML requirements are lodged in the construction industry. In particular, the research initiatives’ objective, importance, and findings have been described, thus outlining the nature of AI and ML development in the construction sector. Looking at the collected data, we identify recurrent patterns, practices, and innovations in AI and ML within construction.

The application of AI in analyzing air pollutants, workforce safety, CPS, and construction materials has led to significant advancements in sustainability and performance optimization. For instance, studying the impact of incorporating waste tire rubber into concrete found that AI models, particularly those using ANN, were able to accurately predict how the size and content of rubber affect the compressive strength of concrete. Moreover, it is also seen that the findings revealed that as the maximum size of the rubber increased, compressive strength decreased more significantly. However, AI-based models like ANN proved adept at maintaining the balance between utilizing recycled materials and preserving structural integrity. Furthermore, the inclusion of supplementary cementitious materials such as metakaolin and fly ash was shown to enhance mechanical properties while reducing environmental impacts, emphasizing AI’s role in promoting sustainability without compromising material performance.

Across multiple studies, ANNs have consistently emerged as powerful tools for various predictive tasks in construction. When predicting the compressive strength of modified mortars, models using ANN demonstrated a high degree of accuracy across diverse datasets, which tend to *R*^2^ as high as 0.96. This predictive capability extends to estimating the effects of different materials and environmental factors, as well as predicting concrete strength over various curing times, highlighting the versatility and power of ANN models (Anumba and Messner, 2020; Abdalla and Mohammed, 2022; Mohammed et al., 2020; Abdalla and Salih, 2022; Mawlood et al., 2021).

Likewise, papers focusing on AI and ML for construction forecasting and decision-making bring attention to how AI optimizes construction activities. For example, the use of ML models for predicting Tunnel Boring Machine (TBM) performance showed that ANN models and SVM-based models could predict operational parameters with up to 95% accuracy. Similarly, it is noted that when applied to deep foundation bearing capacity, optimized SVM models enhanced predictions with high precision (*R*^2^ values up to 0.99), showcasing AI’s ability to reduce costs, improve timelines, and ensure reliability in construction operations.

Hence, in terms of predicting compressive strength, studies leveraging ML techniques like ANN, Multi-Expression Programming (MEP), and Multivariate Adaptive Regression Splines (MARS) were able to estimate the model’s behavior on cement mortars modified with additives. The findings consistently highlighted ANN’s superior performance compared to traditional statistical models, particularly in contexts where materials like metakaolin or fly ash were used. These studies confirmed that ANN models outperformed other techniques, such as Non-Linear Regression (NLR), in achieving lower RMSE and MAE.

Consequently, an important contribution to AI in construction safety comes from research on using optimized SVM combined with evolutionary random forests to predict back-break caused by blasting operations. Moreover, the model demonstrated a strong ability to forecast blasting effects, achieving greater predictive accuracy compared to conventional models and further demonstrating the reliability of AI-based tools for complex construction scenarios.

Additionally, AI has been effectively used in ensuring construction safety. Studies utilizing ANNs to predict soil and foundation stability or to optimize grouting processes with polymers, illustrated how these tools help forecast material behavior under varying conditions. The use of AI algorithms has led to improvements in safety protocols and operational efficiency by providing real-time predictive insights into construction material performance (Jaf et al., 2024; Mahmood and Mohammed, 2022; Omer et al., 2024; Wang et al., 2022; Yu et al., 2021).

Thus, by aggregating these study’s results, we sum up the contributions of those researchers and underline the role of research in many significant fields which emphasize the need to enhance environmental sustainability, safety, and efficiency. However, it must be highlighted that effectively integrating AI and ML has a tremendous positive impact on environmental sustainability by achieving high efficiency in resource consumption and eliminating waste. It is noted that air pollution contributes to climate change, harms ecosystems, and poses severe health risks to humans, including respiratory and cardiovascular diseases. Therefore, monitoring air pollutants is essential for mitigating these adverse effects.

Similarly, these technologies also increase safety requirements because every process is analyzed and controlled with the help of predictive analysis and automation to eliminate risks and incidents. Also, monitoring health parameters within the construction work is crucial due to the high-risk nature of construction work. It is seen that the construction workers are often exposed to hazardous conditions that can lead to accidents and long-term health issues. By integrating health monitoring systems, potential risks can be identified and mitigated in real time, ensuring a safer working environment. Several studies indicate that the integration of AI and ML in the field is used to enhance productivity and expand operations in areas of forecasting and decision-making.

Therefore, this study reveals the possibilities that AI and ML have brought to transform the construction industry, how such transformations have written history, and how they have opened paths for other works. The increasing field of AI and ML applications within the construction sector offers great potential for altering industrial practices, promoting sustainability, and improving overall performance. This study is vital for understanding the complexities of this revolutionary nexus, as it delivers an extensive summary of previous research efforts, emphasizing important patterns and frameworks as a road map for additional studies. With increasing application of AI and ML techniques in the construction industry, this study is well-positioned to educate researchers and professionals toward a future where technology underpins safe, effective, and sustainable construction methods. This survey has real-world implications for all participants in the construction sector.

Conclusively, to the very best of our knowledge, this is the first survey study that emphasizes AI and ML techniques in the construction sector, specifically on pollutants, construction materials, CPS and physiological signals. A vital part of this analysis is examining the survey’s uniqueness and innovation as a contribution to the scientific conversation. Despite the growing popularity of AI and ML in construction, there is a severe lack of comprehensive surveys that compile research results and provide a systematic overview of the topic. This review aims to close this critical gap by providing a comprehensive summary of existing knowledge, identifying essential research gaps, and suggesting directions for future investigation.

This study is organized as follows. Section 2 presents a literature review to comprehensively understand the existing research and developments related to pollutants, CPSs, construction materials and physiological activity monitoring within the construction industry. Section 3 summarizes key studies, highlighting their methodologies, datasets, results, and limitations. Hence, these studies cover various applications, from environmental forecasting to safety management and productivity enhancement. Section 4 highlights the wide range of applications covered by the reviewed studies, from forecasting air pollutant concentrations to estimating concrete performance and automating activity recognition for enhancing worker safety. Finally, Section 5 draws conclusions about the proposed study in terms of findings, recommendations, limitations, implications, summary of novelty and contribution.

## 2 Literature review

We collected studies and data from various sources (PubMed, Scopus, WoS, MedRxiv, ArXiv, Google Scholar, and Scispace) to discover relevant publications.

In particular, we have considered, besides the primary term “AI and ML Technique in Industry 4.0,” multiple synonyms: “Construction Industry 4.0,” “Air pollutant in Construction Industry,” “Physiological Signals of Workers in Construction Industry,” “Air Pollution and Hazard prevention through AI in Industry,” “Cyber Physical Systems,” “Cybersecurity” and “Compressive strength of construction materials.” These terms have been used in combination with “artificial intelligence,” “machine learning,” “deep learning,” “industry revolution 4.0,” and “Digital Industrial Transformation.”

The search yielded substantial literature (150 papers selected from PubMed, Scopus, and Google Scholar, including only two documents from Scispace), including research articles, review articles, case studies, and reports. Among these articles, we selected all the contents about the application of AI methods to construction site and ended up with 36 papers. The criteria used to select the papers were:

- Relevance of the topic: we selected only papers with an innovative approach to AI. Accordingly, we discharged papers with fundamental statistical analysis.
- Completeness and significance of the results: we selected the papers where AI is used to achieve some critical result, removing those papers where AI was only discussed and not a clear result was obtained.

**Figure 2:**
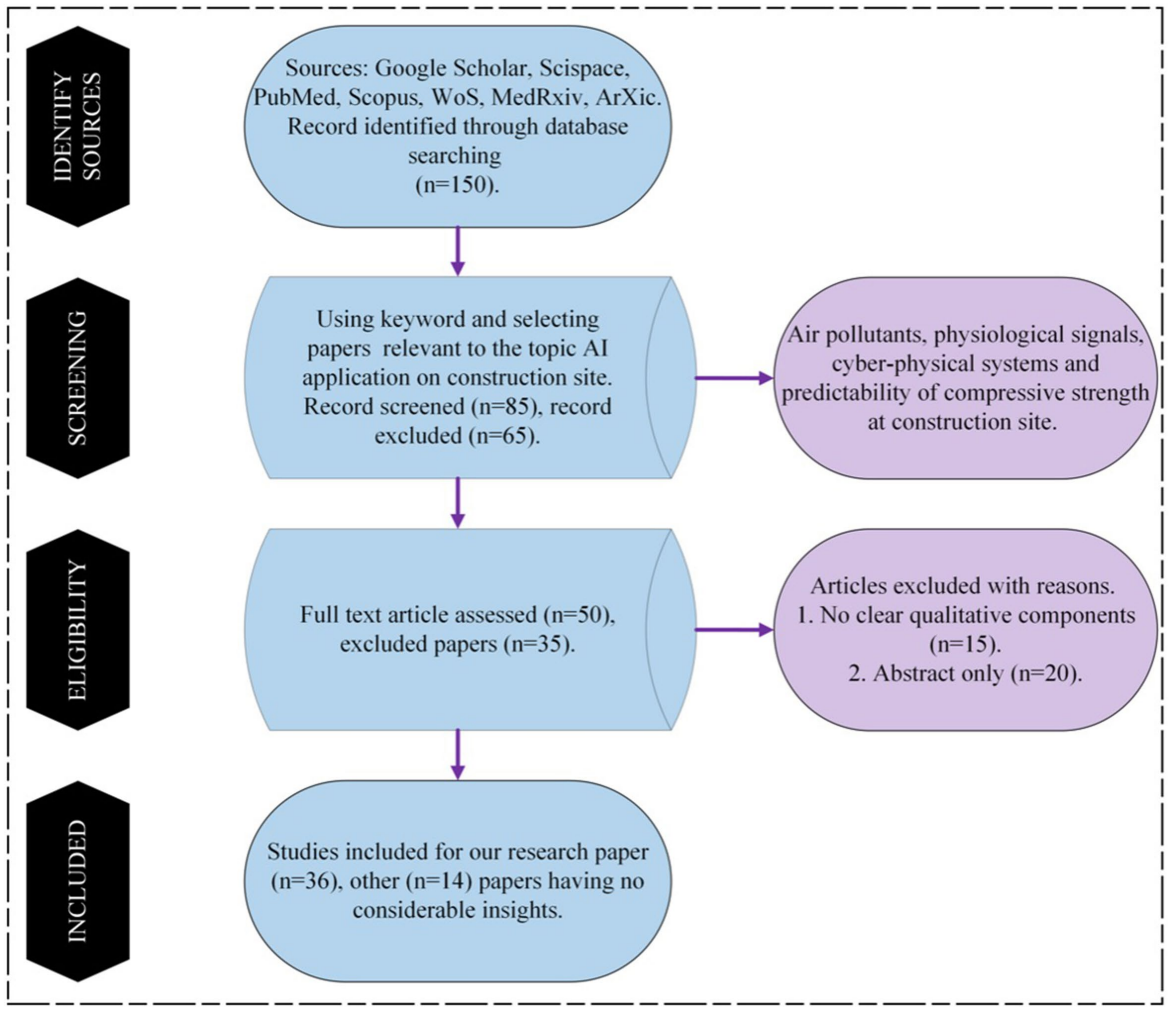
Flowchart of the research approach.

Figure 2 shows the flowchart of the steps performed during the preparation. In the rest of the paper, we first analyze the literature in Section 2.1, where ML and DL models are developed on air pollutants and construction materials. In Section 2.2, the studies show how these AI methods have been applied to workers’ activity. In Section 2.3, the studies conducted on CPS have been discussed and finally, in Section 2.4, the studies included with the prediction of compressive strength of the construction material have been taken into consideration.

### 2.1 AI techniques applied on air pollutants and construction materials

This section presents recent research studies utilizing traditional and novel AI methods to detect air pollutants and features of construction materials. The authors start by applying AI techniques to pollutants and concrete in the construction industry. Accordingly, Table 1 summarizes the reported contributions.

**Table 1:** AI research contributions for predicting pollutants and features of construction materials in construction site.

Authors	Methods	Research objective	Inputs	Results
Al-Janabi et al. (2019)	RNN with PSO	Prediction of the concentration of six air pollutants (PM2.5, PM10, NO_2_, CO, O_3_, and SO_2_)	Data of air pollutants collected, preprocessed and normalized	Average sMAPE over the selected time frame is between 4.34 for SO_2_ and 12.93 for PM2.5
Mastromatteo and Amelio (2024)	LSTM	Prediction of the diffusion level of PM2.5	Environmental features, i.e., drew point, temperature, pressure, wind direction, wind speed, snow and rain levels, past levels of PM2.5	RMSE and *R*^2^ are, respectively, 43.79 and 0.77 in predicting PM2.5 levels 6-h in advance
Baduge et al. (2022)	ANN, SVM, GANs, VAEs, CNNs	Implementation of AI in architectural design, health and safety, security, and emission controls	Data collected by IoT devices	Impact of energy and emission on construction and control through AI
Liu et al. (2022)	ANN with backpropagation	Prediction of dust particle concentration and pollutant	Dust emission characteristics using air characteristics: temperature, pressure, humidity and velocity of wind	Concentration of dust in different working areas under different environmental conditions: *R*^2^ is 0.98, 0.99, 0.97, and 0.97 in the foundation area, rebar processing area, concrete rebar area, and road area of the foundation stage, respectively
Asadi et al. (2014)	Levenberg–Marquardt ANN and NF model	Prediction of the concentration of NO_2_	Traffic count, air humidity, temperature and speed, solar radiation	From the NF model: *R*^2^ is 0.97, 0.95, and 0.94 on training, validation and test sets, respectively
Jassim et al. (2017)	Multilayer perceptron ANN	Hourly energy consumption and CO_2_ emissions of different models of Caterpillar excavators in distinct earthwork conditions	Digging depth, total cycle time, bucket size and payload, load factor and horsepower	*R*^2^ is 0.997
Hendi et al. (2017)	Feedforward ANN with backpropagation	Estimating the concrete specimens’ mass loss and volume loss	Experimental data of 14 concrete mix designs subjected to H₂SO₄ medium data	Loss in mass and volume of the specimen. RSME is 0.44 for mass loss and 1.18 for volume loss
Kwon and Song (2010)	ANN with backpropagation	Determining the change in porosity due to carbonization estimating the diffusion coefficient of CO_2_	Cement content, water-cement ratio, volume of the aggregate, and relative humidity	Maximum error between estimated and experimental data is 6.3% for the estimation of the depth of the carbonation under a range of relative humidity levels
Shahnavaz and Akhavian (2022)	ANN, RT, RF, linear regression	Determination of the emissions of CO, NO_X_ and CO_2_	Different measurements from accelerometer and gyroscope sensors	Emission of air pollutant by utilization of different algorithms. RF algorithm: *R*^2^ is 0.94, 0.91 and 0.94, while normalized RMSE is 4.25, 6.42, and 5.17 for predicting CO, NO_X_ and CO_2_, respectively
Milivojević et al. (2023)	Fundamental statistical and correlation analysis	Determination of the pollution level at the construction site	Air pollutants (NO_2_, PM2.5, and PM10) and meteorological parameters (wind speed and direction, humidity, pressure, and temperature)	Average concentrations of dust emission are 16.42 μg/m^3^ for PM10 and 8.37 μg/m^3^ for PM2.5. Construction activities significantly increased PM10 and PM2.5 concentrations downwind by approximately 70 and 35%
Farahzadi and Kioumarsi (2023)	Genetic algorithms, regression models, ANN, SVM, RF, DT	Finding AI and ML techniques that have contributed to reduce CO_2_ emissions in construction	78 papers selected from an initial pool of 678 identified papers through a systematic review process	ANN models showed promising results in predicting CO_2_ emissions with a MAPE below 10%

Al-Janabi et al. (2019) introduced an intelligent forecasting model for air pollutants’ concentrations in the next two days by using DL techniques, mainly a Recurrent Neural Network (RNN), enhanced by a Particle Swarm Optimization (PSO) algorithm. This model is named the Smart Air Quality Prediction Model (SAQPM). The AI method adopted in the paper is based on unsupervised learning with an extended short-term memory network, which was formerly improved with a functional PSO algorithm to predict the concentrations of numerous types of air pollutants. The dataset includes data on six kinds of air pollution (PM2.5, PM10, NO_2_, CO, O_3_, and SO_2_) collected from multiple monitoring stations, which was then preprocessed to handle missing values and standardized using the MinMaxScaler method. The data has been normalized to a range (0,1). Furthermore, a ten-fold cross-validation principle was applied to split the dataset into training and testing sets in the experimental setting. The functional PSO algorithm aided in tuning hyperparameters to build an effective predictor. The predictor’s performance was assessed for each station by average symmetric Mean Absolute Percentage Error (MAPE) over 25 days, based on hourly readings of pollutant concentrations for up to 30 days. The metric evaluation uses the sMAPE over the selected time frame, which shows average values in predicting air pollutant concentrations between 4.34 for SO_2_ and 12.93 for PM2.5.

Also, Mastromatteo and Amelio (2024) developed a novel DL framework using RNNs to monitor and predict the spread of air pollutants over time, specifically PM2.5, on construction sites. Input data are environmental features acquired from sensors positioned in the construction site, i.e., drew point, temperature, pressure, wind direction, wind speed, snow and rain levels. A Long-Short Term Memory network (LSTM) was pre-trained to predict future PM2.5 levels from the known environmental conditions and past levels of PM2.5. The methodology included four steps: (i) data preprocessing, (ii) model training, (iii) model testing, and (iv) model deployment on construction site. The dataset used for pre-training the model included 43,824 hourly timesteps of environmental features and PM2.5 over five years. Data was pre-processed through visual inspection, binary transformation, handling missing data, box plots and statistical analysis of variables, data aggregation, analysis of cross-correlation and autocorrelation, verification of data stationarity, data normalization and standardization. For the analysis, data was aggregated in 6-h blocks, and divided into training, validation and test sets (70, 10 and 20%, respectively). Also, a random search procedure was performed to optimally set the model hyperparameters, resulting in a number of units of 100, dropout rate of 0.1, learning rate of 0.01, batch size of 32, and Adam optimizer. For each trial of the random search, a 3-fold cross validation was performed and average performance measures were computed. The LSTM model predicted PM2.5 levels 6-h in advance with values of RMSE and *R*^2^ which are, respectively, 43.79 and 0.77 on the test set. It indicates very promising pre-training results.

Finally, Baduge et al. (2022) provided a comprehensive overview of the application of AI, ML and DL approaches in various facets of the building and construction Industry 4.0. The review paper introduced the use of ANN and SVM to predict the mechanical properties of construction materials like concrete, steel, and timber. Generative Adversarial Networks (GANs) and Variational Autoencoders (VAEs) were also used for automated architectural generative design. Convolutional Neural Networks (CNNs) were adopted for other architectural design tasks, like house style recognition and indoor scene synthesis. Other AI techniques were also applied in structural design and analysis, offsite manufacturing automation, construction management, intelligent building operation, detection of hazardous chemical pollutants and sustainability. The article reviewed various datasets used in different AI applications within the construction industry, e.g., design data, material property data, sensor data, seismic data, construction site data, and big data from offsite manufacturing. Overall, the review highlights the transformative potential of AI/ML/DL in the construction industry, paving the way for more intelligent, efficient, and sustainable construction practices.

Regarding the application of ML techniques, Liu et al. (2022) proposed a dust concentration model with construction phases and site climate conditions. It has been developed to fill the lack of a sample model for predictions concerning dust concentration for further implementation of measures to minimize it. A backpropagation ANN model was used in the study to model and forecast the concentrations of dust emanation in construction site areas under diverse conditions using the results of dust generation monitoring. Applying the data obtained from the dust generation monitoring, the backpropagation ANN model was used to simulate and estimate the emanation concentrations of the dust in the site areas due to the influence of the pre-set condition on the construction site. The data considered in the work are the dust emissions received during two stages of a residential construction object (laying the foundation and constructing a load-bearing structure). The data concerns such factors as temperature, humidity, and velocity of the wind. Hence, the given data set was split into training and test data sets to evaluate the model’s efficiency. There were 60 samples collected in total, whereby the first set of 50 samples was proposed for training purposes, while the last 10 samples were proposed for testing purposes. The conclusion and observation of this paper also synthesize that the application of the backpropagation ANN model is helpful in predicting the different alterations of the dust concentration in the different work areas and climates. The efficacy of the model was established through a comparison of the results with those of the conventional regression models, thus evaluating the viability of the model. The results in terms of *R*^2^ between measured and predicted dust emission concentration of different work areas of the foundation stage show a high fitting degree, with values of 0.98, 0.99, 0.97, and 0.97 in the foundation area, rebar processing area, concrete rebar area, and road area of the foundation stage, respectively.

A backpropagation ANN model was also adopted by Asadi et al. (2014) to predict NOx concentration in the air as a function of traffic count and climatic conditions before and after applying titanium dioxide (TiO_2_) on asphalt pavements. The study employed ANN models and Neuro-Fuzzy (NF) models to estimate NO_x_ concentrations, providing early warnings. The study adopted two ML methods. The feed-forward neural network was trained with the Levenberg–Marquardt algorithm. Also, the NF model was combined with the ANN, employing a hybrid algorithm that integrates least squares and backpropagation gradient descent methods. The data set was obtained from a field investigation at Baton Rouge, LA, where an aqueous solution of nano-TiO_2_ was sprayed on asphalt concrete. Therefore, the proposed NF model successfully fitted the NO_x_ measurements better than the ANN model and gave a better-enhanced *R*^2^ measures in all steps of the training, validation, and testing processes, which are 0.97, 0.95, and 0.94, respectively. The analysis established that both models are viable for estimating NOx concentrations and can contribute to the scientific foundation of pollution prevention.

Unlike the above works, Jassim et al. (2017) proved the applicability of the ANN model to predict the hourly energy consumption and CO_2_ emissions of different models of Caterpillar excavators in distinct earthwork conditions. The study employed a multilayer perceptron ANN with a sigmoid activation function and backpropagation training technique for the analysis. As for the data, 5,092 operation modes for 25 models of Caterpillar excavators are included in the dataset and obtained from the Caterpillar manual. Specifically, a 9:1 ratio was applied wherein 9 parts were used for training and 1 part for testing. The performance of the model was checked based on the minimum square error and the *R*^2^ measure. The proposed model proved to be very accurate in predicting hourly energy and CO_2_ consumption with *R*^2^ values of 0.997%. Therefore, the study provides the logical conclusion that the ANN model is sufficient for generating forecasts of energy and CO_2_ consumption. It demonstrates its possibilities of expansion for predicting energy consumption and greenhouse gas emissions using the discrete event simulation data from constructions.

Furthermore, Hendi et al. (2017) studied the resilience of the concrete regarding sulfuric acid corrosion, which is prevalent in sewage systems. The concern of the research is on the effectiveness of incorporating glass powder and micro-silica in concrete mixes with reference to durability against acids. Accordingly, an ANN model was applied for estimating the concrete specimens’ mass loss and volume loss. The ANN model used for the study was a feedforward neural network with backpropagation used in the error computation. The model calculates mass loss and volume loss at a given time of treatment. The dataset includes experimental data of 14 concrete mix designs subjected to H₂SO₄ medium data, whose 60% was adopted for training the model, 20% for validating the model, and the final 20% for testing the model. The ANN’s short-term mass loss forecast was promising, with a RMSE of 0.44 and volume loss with an RMSE of 1.18; the results show high prediction accuracy. A study conducted in an acidic environment showed that glass powder and micro-silica help considerably increase concrete’s durability. The findings established that the concrete with a greater content of micro-silica and glass powder provides better performance in sulfuric acid regardless of the lower compressive strengths.

The ANN model was also employed by Kwon and Song (2010), which analyzed the carbonation behavior in concrete structures through the creation of a numerical technique based on ANN. The latter, adopting a backpropagation method for training, was employed to estimate the diffusion coefficient of CO_2_, thus extending the chances of enhancing its efficiency over that of the expensive experimental approaches. The input parameters are cement content, water-cement ratio, volume of the aggregate, and relative humidity. The data collected in the analyzed study is based on experimental data. The maximum error between estimated and experimental data is shown to be 6.3% for the estimation of the depth of the carbonation under a range of relative humidity levels. This work describes a feasible numerical analysis based on ANN for forecasting the carbonation kinetics in concrete structures.

Another different approach was proposed by Shahnavaz and Akhavian (2022). The work aims to develop and deploy a ML framework to predict emissions from heavy construction equipment. Different ML algorithms have been utilized, ANN, Regression Trees (RT), Random Forest (RF), and linear regression. Data was collected from a Caterpillar 305D CR excavator performing real-world construction work. The input data includes different measurements from accelerometer and gyroscope sensors, while the output data are the emission levels of CO, NO_X_, CO_2_, SO_2_, and CH_4_ recorded using a portable emission measurement system. Additionally, 70% of the dataset was used for training and 30% for testing. The RF model demonstrated the best performance with *R*^2^ equal to 0.94, 0.91 and 0.94 and normalized RMSE of 4.25, 6.42, and 5.17 for predicting CO, NO_X_ and CO_2_, respectively. Hence, the study successfully predicted the emissions of heavy construction equipment using ML models trained on sensor data, achieving high accuracy and demonstrating the potential for broader application and future research enhancements.

Retrospective research conducted by Milivojević et al. (2023) involved implementing a distributed sensor network to collect real-time data on air pollutants and meteorological parameters to manage construction operations effectively to reduce environmental impact. The dataset comprised real-time measurements of air pollutants (NO_2_, PM2.5, and PM10) and meteorological parameters (wind speed and direction, humidity, pressure, and temperature). The possibility of using ANN for predictive modeling has been mentioned but excluded due to the very low correlation between PM particle concentration and meteorological parameters. The study is interesting but involved fundamental statistical and correlation analysis without using a validation strategy like K-fold cross-validation. The study found high dust emission levels at the construction site. Average concentrations were 16.42 μg/m^3^ for PM10 and 8.37 μg/m^3^ for PM2.5. Construction activities significantly increased PM10 and PM2.5 concentrations downwind by approximately 70 and 35%, respectively. PM2.5 levels posed a far more significant health hazard due to higher values than prescribed daily limits.

Farahzadi and Kioumarsi (2023) proposed a survey work on how AI and ML technologies have contributed to reducing CO_2_ emissions in the construction sector. The study identifies and discusses various ML techniques used in the literature, including genetic algorithms, regression models, ANN, SVM, RF and Decision Trees (DTs). The dataset for this review consists of 78 papers selected from an initial pool of 678 identified papers through a systematic review process. The study follows a systematic review methodology, with the following steps: (i) database selection, (ii) filtering, (iii) initial identification of 678 papers. Bibliographic and content analyses were conducted on the selected documents to categorize and analyze the significance. Most studies in this review paper focused on sustainable materials and components design/production. ANN models showed promising results in predicting CO_2_ emissions with a MAPE below 10%. The paper is crucial in optimizing and predicting CO_2_ emissions reduction in the construction sector using AI.

### 2.2 AI techniques applied to physiological activity

This section presents recent research studies utilizing traditional and novel AI methods to detect physiological activity. The authors start by applying AI techniques to worker activity in the construction industry (Table 2).

**Table 2:** AI research contributions for monitoring of physiological signals in construction site.

Authors	Methods	Research objective	Inputs	Results
Mäkela et al. (2021)	XGBoost, LDA, LR, RF, SVM	Enhancing safety and improving work conditions and health	Utilization of wearable sensors like IMU to get real-time data	Comparison of datasets of workers’ activities based on ML algorithms and definition of their work’s safety and ergonomics. The model accuracy is 89% for six classes and 78% for sixteen classes
Aryal et al. (2017)	SVM, DT	Establishing methods for assessing fatigue	Heart rate, brain wave, and temperature sensor data	Thermoregulation from the temple is more valuable than heart rate. There was a ten-fold cross-validation with 82% accuracy of heart rate and temperature sensors. The accuracy was 59% and 79%, considering alone the heart rate and the temple temperature respectively
Akhavian and Behzadan (2018)	ANN, SVM, LR, DT and KNN	Investigating, detecting, and classifying construction workers’ activities	Data captured from the smartphone’s built-in gyroscope and accelerometer	Comparison of data-driven and static simulation values with ten-fold cross-validation. ANN model attained an accuracy of 90.74%
Karataş and Budak (2021)	LR, SVM, DT and KNN	Build AI models to recognize activities in construction work and effectively utilize project management and control	Data collected by axis accelerometer, gyroscope, and magnetometer	The best prediction was obtained with the SVM algorithm, which had an accuracy of 90%. In other algorithms, 887% and 80% accuracy values were achieved with the KNN and DT algorithms, respectively
Sherafat et al. (2020)	SVM, ANN, DT, KNN, LR, RF, Naïve Bayes, RNNs	Investigation of the performance-based issues and productivity rate at construction job sites	Location tracking, activity recognition tracking, and performance monitoring	Providing a comprehensive review and comparison of different conducted studies
Akhavian and Behzadan (2016)	SVM, DT, LR, ANN	Understanding the behavior and surrounding context of construction workers	Data collection by smartphone built-in and sensor logging apps	Low-cost pervasive construction workers’ activities recognition system proposed. The model attained with ten-fold cross-validation accuracy of 87–97% (user-dependent), and 62–96% (user-independent)
Al Jassmi et al. (2020)	ANN, SVM, KNN	Proposing a novel approach for construction workers to capture physiological signals using remote and automatic activity recognition	Data collected by Zephyr Bioharness™ and Empatica E4 wristband	The blood volume pulse (BVP), respiration rate (RR), heart rate (HR), GSR and skin temperature (TEMP) values were extracted through the sensor; hence, the ANN achieved an 88% accuracy level with ten-fold cross-validation
Ouyang et al. (2023)	KNN, SVM, RF and LDA	Developed a predictive method to analyze fatigue by using ECG and GSR	HR variability, GSR	Findings indicate that ECG sensors used alone or in combination with GSR sensors can be applied to monitor construction workers’ inattention on job sites. Model accuracy with KNN and HRV features: 88.33%, GSR features: 76.67%, and combined features (SVM): 96.67%
Joshua and Koshy (2011)	DT, multilayer perceptron ANN and backpropagation learning	Recognition of activities of workers to enhance productivity at the construction site	Accelerometer data sampling	Different classifier techniques were analyzed to evaluate the best classifier for activity recognition. The model attained 80% accuracy with accelerometers attached at both sides of the waist
Peddi et al. (2009)	Feedforward ANNs with backpropagation learning	Recognition of human poses images based on effective, ineffective and contributory work	Initial data acquired from wireless real time productivity measurement capturing the images of the activities using a video camera	Determine two parameters: image processing and productivity at construction site. The model attained 85% of accuracy

Mäkela et al. (2021) performed a study to utilize working conditions and safety in labor-intensive fields, mostly in construction, by implementing IoT technology and wearable sensors for human activity recognition. The classifiers included RF, extra trees, XGBoost, Linear Discriminant Analysis (LDA), SVM and Logistic Regression (LR), which were used in this study to identify human activities. The classifiers were trained using the standard configuration because fine-tuning of hyperparameters did not significantly affect performance. The data was further divided into training, validation, and testing sets based on the Leave-One-Subject-Out (LOSO) evaluation which reflected the model’s performance with different perspective. The dataset aims to benchmark Human Activity Recognition (HAR) and improve occupational safety and wellbeing in professional construction settings. The experiment introduced the VTT-ConIoT dataset, which contains data from 13 users performing 16 different construction-related activities, collected from accelerometers which are placed on hip, upper arm and back shoulders. This study attained classification accuracy of 89% in the six-class setup and 78% in the sixteen-class setup. The results indicated the potential for generalization to different individuals and suggested the usefulness of a sensor-based approach to recognize recommended and non-recommended activities in construction settings.

Furthermore, Aryal et al. (2017) defined a novel technique for real-time monitoring of physical fatigue in construction workers using heart rate monitor, infrared temperature sensors and EEG sensors. The aim was to improve the safety trials, which tends to strengthen work-rest schedules. Based on physiological data, boosted tree classifiers were employed to predict physical fatigue levels. The dataset comprised data collected from 12 participants wearing sensors for 20 min at rest, recording baseline heart rate, skin temperatures, and EEG waves. Subjective fatigue levels were recorded using Borg’s Rating Of Perceived Exertion (RPE) scale. The experimental setting involved participants wearing sensors and resting for 20 min to record baseline physiological data. The material handling task, which broke every 50 trials for the completion of the Psychomotor Vigilance Test (PVT) test, was performed. Moreover, different classification algorithms were tested, and a ten-fold cross-validation approach was used for assessment. The accuracy achieved by the boosted tree was 82% with all extracted features like heart rate and temperature sensors yielded in predicting physical fatigue levels, on the other hand with monitoring from the temple showing promising results of 79% accuracy. On the other hand, monitoring of the only temple temperature showed promising results of 79% accuracy whereas relying on the only heart rate the accuracy was 59%.

By contrast, Akhavian and Behzadan (2018) addressed the challenges of input modeling in construction simulation models by applying smartphone sensors, i.e., accelerometer and gyroscope, to track construction worker’s body movement activities. The dataset consisted of timestamped sensor data from multiple construction workers performing various activities such as sawing, loading, hauling, unloading, hammering, and turning a wrench in a controlled outdoor environment simulating a construction job site. Moreover, the experiment tests were performed on 30 cycles, and then recordings were observed for each activity. Two simulation models that were based on the Activity Cycle Diagram (ACD) concerning different probability distributions for activity periods were developed. The study focused on supervised learning approaches to forecast workers’ activities, testing various classifiers like ANN, SVM, K-Nearest Neighbor (KNN), LR, and DTs. The performance was determined by using ten-fold cross-validation after many trials, with ANN showing the highest accuracy, followed by KNN. For the data-driven simulation model, which was composed of real-time sensor data, it was shown that the ANN outperformed the other techniques, with accuracy of 90.74%, which depended on the estimated activity intervals.

Additionally, Karataş and Budak (2021) improved labor control and management efficiency in construction projects by developing AI models for activity recognition. The study used sensor data to automatically recognize construction activities performed by laborers on construction sites. The AI-adopted method involved collecting data from 3-axis accelerometer, gyroscope, and magnetometer sensors worn by laborers. The raw data underwent preprocessing, including segmentation and statistical feature extraction. The dataset used in the analysis consisted of 76,080 data points collected from activities such as logging, carrying, surfacing, vibrating, and waiting. ML algorithms such as LR, SVM, DT, and KNN were utilized for training and modeling. The experimentation analysis adopted ten-fold cross-validation to minimize bias. The SVM algorithm attained a maximum accuracy of 90%, followed by the KNN algorithm with an accuracy of 87%. In contrast, the LR and DT algorithms achieved approximately 80% accuracy. Precision, recall, and F1-score values also highlighted the superior performance of the SVM algorithm in predicting construction activities.

A review work was proposed by Sherafat et al. (2020) with the integration of different ML and DL methods. The goal was to improve productivity in construction by monitoring workers and equipment. In the paper, the workforce monitoring system was described as composed of four levels: (i) tracking location, (ii) recognizing activities, (iii) monitoring activities, and (iv) assessing performance. The data sampling was done by built-in sensors of smartphone, i.e., accelerometer and gyroscope. The study integrated mechanized activity recognition techniques, focusing on ML algorithms like SVM, ANN, DTs, KNN, LR, and RF, emphasizing ANNs and DL, remarkably Naïve Bayes, CNNs and RNNs. The paper included studies on automated activity recognition regarding construction categorized into audio-based, kinematic-based, and computer vision-based techniques. The experimental setting investigated automated activity recognition, focusing on proof-of-concept stage experiments and discussing trials such as limited ground-truth data. The paper focused on the need for commercially applicable methods to detect and recognize equipment activities accurately, highlighting the importance of different approaches and asserting the limits of automated activity recognition methods in construction sites.

Akhavian and Behzadan (2016) developed advanced construction project management, which automated the identification of workers’ logs using mobile phone sensors, i.e., accelerometer and gyroscope. The data captured body movement using simulated construction activities (e.g., loading, sawing, dumping). ML approaches were used to accurately determine data from smartphone sensors and then recognize the different construction tasks, reflecting the improvement of productivity and safety management. The classifiers used in this research are ANNs, DTs, LR, SVM and ensemble models. The dataset comprised data collected from smartphone sensors and logging apps on mobile platforms. The study employed a ten-fold stratified cross-validation approach, where 90% of the data was used for training and 10% for testing. The study defined the fine-tuning model parameters based on regularization factors to optimize performance. The investigation demonstrated high accuracy rates of over 90% across various activity categories, with classifiers like ANNs, which accomplish accuracies of 87–97% for user-dependent activities and 62–96% for user-independent categories.

Al Jassmi et al. (2020) proposed a method for automating construction worker performance monitoring using wearable sensors to gather physiological data, enabling remote and automatic activity recognition. The study addressed classification challenges in distinguishing between productive and nonproductive activities and identifying specific types of productive tasks based on physiological signals. The proposed method involved training various ML classifiers using physiological signals such as blood volume pulse, respiration rate, heart rate, Galvanic Skin Response (GSR), and skin temperature. The dataset used in the analysis was obtained through an Android mobile application designed for data fusion, integrating real-time physiological readings from sensors like Zephyr Bioharness™ and Empatica E4 wristbands via Bluetooth. The experimental setting involved a pilot study with three pre-fabrication stone construction workers. The dataset underwent a ten-fold cross-validation split for training and testing the ML classifiers. The study achieved promising results, with an accuracy of up to 88% in activity recognition using an ANN classifier. Two classification problems were addressed: classification of various activities as productive and nonproductive for identifying the productive tasks and activities based on a nonproductive task (binary model) and for accomplishing some productive tasks based on a (multi-class) model.

Furthermore, Ouyang et al. (2023) proposed a predictive model based on electrocardiogram (ECG) and GSR sensors to assess the inattention of construction workers due to physical fatigue. The study determined the relationship between workers’ physiological features and attention levels during attention-demanding tasks. For the computational models, several supervised learning techniques, including SVM, KNN and RF, as well as LDA, were used to predict cognitive states, which are based on ECG and GSR sensor data features. These models aim to predict the workers’ attentional states, with Heart Rate Variability (HRV) features derived from ECG signals and skin electric activity features derived from GSR signals. The data set was composed of 30 participants who completed cognitive tasks twice. The first practice was under non-fatigued circumstances. In contrast, the second one was done after the physical exertion of fatigue. Additionally, the experimental setup described each subject performing cognitive tasks twice, which were randomly split into two groups to avoid order effects. Hyperparameters were selected using grid search, and LOSO was used for model evaluation. Results showed that using HRV features alone achieved an 88.33% prediction accuracy with the KNN algorithm, while GSR features alone achieved 76.67% accuracy, also with KNN. Combining HRV and GSR features enhanced accuracy by 96.67% through the SVM algorithm.

A different approach proposed by Joshua and Koshy (2011) presented a task of accelerometer-based activity classification to automate work sampling on construction sites (brick lying on an uncompleted wall of 450 mm in height). The main objective was to improve safety, productivity, and quality control. The study aimed to evaluate different classification techniques, focusing on masonry activities combined with the multilayer perceptron ANN, which reflected good results. Data sampling was collected from masons evaluated while performing instructed and uninstructed activities with accelerometers attached to their waists. Ten runs of ten-fold cross-validation per classifier were performed and ANOVA tests used for comparing data from sensors that are placed at different positions. Data was segmented into windows of lengths 2, 4, and 4.23 s with a 50% overlap. The multilayer perceptron achieved 80% accuracy in uninstructed mode. It motivated the emphasized segment overlap’s importance for classifier performance and used only the best features to reduce runtime without accuracy loss. The study examined the significance of underscoring data collection mode, feature types, and segment overlap in accelerometer-based activity recognition for construction work-sampling automation.

Finally, Peddi et al. (2009) presented a computerized productivity measurement system for on-site construction using computer vision and AI principles. The system captured real-time construction task images, which extract human poses, categorized them, and utilized an ANN to measure worker productivity. The goal was to expedite construction processes through instantaneous, automated productivity assessment. The study used a neural network-based approach for pose classification. Specifically, it employed feedforward ANNs with backpropagation learning to classify poses as effective, ineffective, and contributory. Data was sourced from the Wireless Real-time Productivity Measurement (WRITE) system, which communicated construction activity image classifications through video camera (having pan, tilt and zooming features) over the Internet. The established algorithms attained an accuracy of about 85%, equivalent to manual methods, offering instant feedback to construction teams and enhancing the efficiency of construction processes. This progression eliminated biases and limitations related to traditional manual procedures, allowing real-time productivity valuation in the construction sector.

### 2.3 AI techniques for cyber-physical systems in construction industry

This section presents recent research studies leveraging the integration of traditional and novel AI methods with CPS methodologies which leads to the enhancement of the effectiveness of Industry 4.0. The authors start by applying AI techniques for CPS to the construction industry. Accordingly, Table 3 summarizes the reported contributions.

**Table 3:** AI research contributions toward CPSs in construction industry.

Authors	Methods	Research objective	Inputs	Results
Moosavi et al. (2024)	Explainable AI techniques	Exploring XAI techniques in manufacturing and industrial CPS, enhancing the transparency and interpretability of AI models in critical applications	Academic and industry publications on AI and XAI applications in industrial CPS and manufacturing	Role of XAI in improving AI reliability and human comprehension in manufacturing systems
Radanliev et al. (2021)	Development for AI use in CPS, focusing on anomaly detection and system resilience	Developing a conceptual framework for AI’s evolving role in CPS, highlighting challenges and transparency in AI-based decision-making	Academic and industry papers from 2010–2020, focusing on AI and CPS in Industry 4.0	Hierarchical cascading framework for transparent AI decision-making in CPS, addressing the challenges of AI evolution
Anumba and Messner (2020)	Implementation of CPS prototypes using sensors	Enhancing construction project operations through implementation of digital models for real-time tracking, monitoring, and control	Data from construction components, structures, and mobile cranes monitored via sensors and tracking systems	Enhanced project control through real-time feedback; prototypes demonstrating CPS applicability in construction sites
Correa and Maciel (2018)	CPS framework based on virtual models using Petri Nets connected to BIM models and hardware sensors	Integrating computational resources with construction processes to improve the efficiency and sustainability of project delivery	Data from real-time construction processes, BIM models, and computational tools like wireless sensors and visualization	Highlighted potential benefits of CPS for intelligent and sustainable construction, ensuring project task completion
Anumba et al. (2010)	Development of models using wireless sensors and data fusion	Integrating BIM and CPS in construction processes, enabling bi-directional communication between virtual models and on-site construction activities	Virtual 3D and 4D BIM models, on-site hardware like sensors and actuators and data from construction progression	Framework for real-time monitoring and optimization of construction processes, bridging BIM and on-site activities

Moosavi et al. (2024) explored the integration of Explainable AI within manufacturing and industrial CPS. The study highlighted that Explainable AI methodologies are crucial for validating that CPSs meet their dynamic operational requirements. The formal representation of requirements, such as process control, predictive maintenance, and fault diagnosis, was achieved using interpretability techniques like Shapley values and Local Interpretable Model Agnostic Explanation. Verification was performed through model-agnostic methods to ensure consistency. Moreover, it allows experts to understand and trust the decision-making process. The paper also discussed the need for lifecycle adaptability in Industry 4.0 systems, emphasizing how CPS requirements must be adaptable corresponding toward changing of environmental aspects.

Also, Radanliev et al. (2021) conducted a detailed survey on the intersection of AI, CPS, and Industry 4.0, focusing on how CPS requirements are represented and verified across their lifecycle. The authors proposed the integration of CPS and IoT into a unified CPS-IoT framework that allows real-time sensing, processing, and actuation. This framework emphasized the need for formal verification of requirements, especially in terms of security and interoperability, given the criticality of Industry 4.0 systems. By utilizing AI-based methods for requirement representation, such as ML algorithms that predict system behaviors, the study highlighted the importance of transparency and explainability to ensure that CPS remains compliant with lifecycle requirements. Verification was facilitated through scenario-based testing and continuous monitoring to adapt to operational changes and emerging cybersecurity threats.

Unlike the previous approaches, Anumba and Messner (2020) examined the development of CPS in construction to facilitate improved consistency between digital models and physical components. The study emphasized the need for bi-directional consistency between components and their digital replicas, underscoring how sensors and actuators provide real-time feedback and actuation for monitoring construction progress. The research introduced the importance of real-time data acquisition, coordination, and adaptability. The use of Petri Nets was suggested for formal requirement modeling, offering a means for structured simulation and optimization of CPS processes. Verification was achieved by assessing consistency in system feedback loops to ensure requirements for safety, functionality, and performance parameters.

Also, Correa and Maciel (2018) proposed a comprehensive framework for the application of CPS in construction, focusing from initial design to construction and subsequent operation. The framework’s core idea was the representation of construction process requirements through the use of Petri Nets, which enabled simulation, validation, and real-time monitoring of progress against expected milestones. The framework emphasized modularity and traceability of requirements, enabling continuous verification through data from sensors and actuators. Additionally, the study addressed the need to balance the complexity of requirement models with their interpretability, suggesting a hierarchical approach for both macro and micro-level activities, ensuring that all operational stages of CPS are covered from design to execution.

Finally, Anumba et al. (2010) delved into the opportunities provided by CPS in integrating virtual models with physical construction processes, with a strong focus on requirement verification and validation (V&V) throughout the project. The study reviewed the state-of-the-art in real-time consistency monitoring between digital and physical assets, identifying how embedded systems, sensors, and data fusion tools contribute to verifying that system requirements are met during each stage of development. To formally represent these requirements, the research advocated for the use of virtual prototyping, 4D Building Information Modeling (BIM), and runtime monitoring to align digital models with on-ground construction. Verification techniques, such as model checking and runtime validation, ensured that the CPS met safety, reliability, and interoperability standards throughout its lifecycle.

### 2.4 AI techniques to predict compressive strength of modeling construction materials

This section presents recent research studies describing the AI methods in terms of predicting compressive strength of construction materials which leads to the enhancement of the effectiveness of Industry 4.0. The authors started forecasting the evaluation regarding the compressive strength of construction materials using AI techniques to the construction industry. Accordingly, Table 4 summarizes the reported contributions.

**Table 4:** AI research contributions to predict compressive strength of modeling construction materials.

Authors	Methods	Research objective	Inputs	Results
Abdalla and Mohammed (2022)	MARS, MEP, ANN	Predicting compressive strength of cement mortar modified with metakaolin	230 data samples of cement mortar mix proportions with varying w/b ratio, sand/binder ratio, metakaolin content	MARS model had highest *R*^2^ of 0.95, and lowest RMSE of 2.89
Mohammed et al. (2020)	ANN, M5P-tree, NLR	Determining model compressive strength of fly ash-modified cement mortar	450 samples of cement mortar with varying fly ash content, w/b ratios	ANN model had *R*^2^ of 0.92, RMSE of 3.15; best model for compressive strength prediction
Abdalla and Salih (2022)	MEP, ANN, M5P-tree	Forecasting compressive strength of cement-based mortar modified by calcium hydroxide	Data samples comprising mix variations up to 45% calcium hydroxide content, w/c ratio, curing age of 1–28 days	MEP model performed best with *R*^2^ of 0.93, RMSE of 2.64, MAE of 2.01
Mawlood et al. (2021)	ANN, linear regression	Predicting UCS and Cc of soils based on various geotechnical properties	253 test samples combined with 350 academic data points; features include Atterberg limits, moisture content, density	ANN had slightly better performance with *R*^2^ of 0.88 and RMSE of 7.25
Jaf et al. (2024)	MEP, ANN, MARS, NLR	Evaluating the impact of waste tire rubber on concrete compressive strength	135 data points on rubber content, size, cement content, water content, and aggregate content	ANN model had *R*^2^ of 0.94, RMSE of 2.54, indicating reliable compressive strength prediction
Mohammed et al. (2020)	ANN, M5P-tree, linear regression, NLR	Predicting compressive strength of cement-grouted sands modified with polymers	Hand-mixed cement-grouted sands with polymer content, sand grain size, w/c ratio	ANN achieved *R*^2^ of 0.91, RMSE of 3.02; BS showed 71% higher strength than ASTM
Omer et al. (2024)	XGBoost, MEP, MARS, ANN	Predicting compressive strength in fly ash- and modified recycled aggregate concrete	295 data points on concrete mixtures with cement, w/b ratio, natural/recycled aggregates, fly ash, and superplasticizer	XGBoost model outperformed others with *R*^2^ value of 0.97, and RMSE of 2.14
Wang et al. (2022)	PCA with ANN-ABC algorithm	Identifying key factors and predicting TBM advance rate	Factors influencing TBM advance rate using PCA to reduce dimensionality	PCA-ANN-ABC model achieved *R*^2^ of 0.96 (training), 0.96 (testing), with RMSE of 0.87 and MAE of 0.65
Yu et al. (2021)	SVM optimized with WOA and MFO algorithm	Predicting back-break caused by blasting operations	Dataset includes 10 inputs affecting back-break magnitude, parameters related to blast design, explosive material, rock mass	SVM-MFO had best performance with *R*^2^ of 0.95 and RMSE of 0.45
Jahed Armaghani et al. (2023)	SVM with different kernels (RBF, polynomial, dot, neural, ANOVA)	Assessment of bearing capacity of deep foundations	141 pile datasets; features include pile geometry, soil conditions, and field test settings	SVM-RBF kernel achieved *R*^2^ of 0.97 (training), 0.99 (testing) and RMSE of 24.8

Abdalla and Mohammed (2022) introduced a novel approach for predicting the compressive strength of metakaolin-modified cement mortar by utilizing a hybrid AI model. It is seen that this model integrates MARS, MEP, and ANN to enhance accuracy in forecasting strength parameters. The study collected 230 data samples, which were used as input for modeling. MARS was employed to develop the primary prediction model due to its ability to handle high-dimensional data and complex nonlinear relationships. Additionally, comparative analysis showed that MARS achieved superior accuracy, with an *R*^2^ of 0.95 and RMSE of 2.89, when tested against MEP and ANN models. Hence, this model’s accuracy demonstrated a significant advancement in automated strength prediction, reducing reliance on manual testing methods and providing an efficient, computerized system for predicting mortar performance in real time.

Also, Mohammed et al. (2020) proposed a robust method for evaluating the compressive strength of fly ash-modified cement mortar using ANN, M5P-tree, and NLR approaches. Moreover, the study utilized a comprehensive dataset of 450 samples with diverse fly ash content, water-to-binder ratios, and curing times. It was processed through ANN to classify compressive strength outputs based on curing ages, fly ash content, and water-to-binder ratios. The ANN model, trained with a backpropagation algorithm, achieved an *R*^2^ of 0.92 and an RMSE of 3.15, showing higher predictive accuracy over NLR with an *R*^2^ of 0.90 and RMSE of 3.35, and M5P-tree with a MAE of 2.45. Finally, by automating the strength classification process and reducing errors associated with manual evaluation, this study emphasized the capability of AI-driven modeling to enhance efficiency in material assessment and processing.

Abdalla and Salih (2022) developed a predictive AI framework for forecasting the compressive strength of cement-based mortar modified by calcium hydroxide. Therefore, employing MEP, ANN, and M5P-tree models, the system leveraged a dataset with varying calcium hydroxide content and curing times, comprising mix variations up to 45% calcium hydroxide content and curing ages of 1–28 days. It is noted that the MEP model demonstrated an enhanced predictive capacity, achieving an *R*^2^ of 0.93, MAE of 2.01 and RMSE of 2.64, compared to ANN and M5P-tree models. Hence, this automated assessment framework facilitated real-time evaluation of material properties, reducing human errors and improving the reliability of strength predictions for modified mortars.

Mawlood et al. (2021) presented a ML-based prediction system for assessing the Unconfined Compressive Strength (UCS) and Compression index (Cc) of soils. Furthermore, using ANN and linear regression models, the study processed a wide array of soil parameters, including Atterberg limits and dry density, to predict UCS and Cc. By using a mix of 253 test samples and over 350 academic data points, the ANN’s feedforward backpropagation approach achieved an *R*^2^ of 0.88 and RMSE of 7.25 for UCS prediction, making it a highly reliable predictive tool, while the linear regression model showed an *R*^2^ of 0.85 and RMSE of 8.12. Hence, the implementation of this AI-based system allows for efficient soil strength evaluation and reduces dependency on extensive manual testing, thereby expediting geotechnical analyses in construction projects.

Jaf et al. (2024) explored the influence of waste tire rubber on the compressive strength of concrete through advanced AI modeling techniques. Moreover, using MEP, ANN, MARS, and NLR models, the study processed a dataset of 135 points, focusing on rubber size, percentage, and curing duration. The ANN model, equipped with a backpropagation learning mechanism, achieved an *R*^2^ of 0.94 and RMSE of 2.54, showing a high degree of accuracy in predicting strength outcomes. Hence, the study effectively demonstrated how AI can streamline the assessment of rubber-modified concrete mixtures, providing instantaneous and accurate evaluations that traditionally required manual testing.

Mohammed et al. (2020) implemented an ANN-based model to predict the compressive strength of cement-grouted sands modified with polymers. The study compared the efficacy of American Society for Testing and Materials (ASTM) and British Standards (BS) in strength assessment, applying a neural network approach to analyze the impact of polymer content and curing conditions. The dataset included features like hand-mixed cement-grouted sands with polymer content, sand grain size, w/c ratio. Tests conducted under ASTM and BS standards revealed that the ANN model achieved an *R*^2^ of 0.91 and an RMSE of 3.02, with compressive strength values 71% higher under BS, outperforming M5P-tree and other regression-based models. Conclusively, this AI-driven system enhances predictive accuracy, provides immediate feedback on material properties, and improves the evaluation process for polymer-modified sands in construction.

Omer et al. (2024) leveraged ML algorithms, including XGBoost, MEP, MARS, and ANN, to predict the compressive strength of recycled aggregate concrete modified with fly ash. Furthermore, a dataset of 295 samples was analyzed to determine the impact of variables like water-to-binder ratio and curing time. The XGBoost model excelled with an *R*^2^ of 0.97 and RMSE of 2.14. Hence, this study underscores the potential of AI models to accurately predict recycled concrete properties, ensuring real-time, precise evaluations that support sustainable construction practices and minimize manual testing efforts.

Wang et al. (2022) presented an AI-enhanced system combining Principal Component Analysis (PCA) with ANN-Artificial Bee Colony (ABC) to predict the advance rate of TBMs. Moreover, the model processed a variety of inputs to optimize the prediction accuracy of TBM advance rate. It is noted that the model could achieve an *R*^2^ of 0.96 for training and 0.96 for testing, with an RMSE of 0.87 and MAE of 0.65. Hence, this innovative approach enabled instant feedback on TBM performance, reducing biases and inaccuracies in manual assessments and enhancing the efficiency of tunnel construction operations over other regression-based approaches.

Yu et al. (2021) applied optimized SVM models, using Whale Optimization Algorithm (WOA) and Moth-Flame Optimization (MFO), to predict back-break occurrences in blasting operations. Furthermore, data was sourced and preprocessed to identify key variables influencing back-break. Dataset included 10 inputs affecting back-break magnitude, parameters related to blast design, explosive material, rock mass. It is seen that the SVM-MFO model achieved an *R*^2^ of 0.95 and RMSE of 0.45, which outperformed SVM-WOA and standard SVM models, whereas sensitivity evaluations confirmed the SVM-MFO’s effectiveness in forecasting back-break magnitude and enhancing blasting operation safety. By providing instant, AI-driven predictions, this study effectively enhanced the accuracy of back-break evaluations, thereby aiding in safer and more efficient mining operations.

Finally, Jahed Armaghani et al. (2023) examined various SVM kernel functions to predict the bearing capacity of deep foundations. Hence, by utilizing a dataset of 141 piles including pile geometry, soil conditions, and field test settings, different models, including Radial Basis Function (RBF), dot, neural, ANOVA and polynomial kernels, were tested. Furthermore, the SVM-RBF model emerged as the top performer, with *R*^2^ values of 0.97 (training) and 0.99 (testing), and an RMSE of 24.8. Conclusively, this AI-based prediction system provides a reliable alternative to traditional pile capacity evaluation, offering real-time results and minimizing human error in foundation design.

## 3 Task description

This section describes the main tasks of the different papers reported in Subsections 2.1–2.4. Tables 1–4 provide a detailed description of the input data and results obtained in the context of air pollutants and construction materials (see Table 1), physiological activity (see Table 2), CPSs (see Table 3), and compressive strength of construction materials (see Table 4).

- Al-Janabi et al. (2019) proposes an intelligent forecaster for air pollutant concentrations using DL techniques enhanced by a PSO algorithm. The study focuses on predicting concentrations of various air pollutants using unsupervised learning with RNNs. The study demonstrates the effectiveness of DL in environmental forecasting.
- Mastromatteo and Amelio (2024) develops a novel framework using LSTM to monitor and predict the spread of air pollutants, specifically PM2.5, on construction sites from known environmental conditions. The methodology includes data preprocessing, model training, model testing, and model deployment on construction sites. The LSTM model predicts PM2.5 levels 6-h in advance with good RMSE and *R*^2^ values which indicate promising pre-training results.
- Baduge et al. (2022) provides an overview of ML and DL techniques in the construction industry, emphasizing their role in project lifecycle management and safety enhancement. The study showcases a range of AI techniques implemented in construction safety and management, highlighting the integration of intelligent vision technologies with AI. It provides valuable insights into AI applications in construction.
- Liu et al. (2022) studies the emission appearances of construction work dust particles using a backpropagation ANN model. The research mainly focuses on assessing and forecasting dust concentrations at construction sites, utilizing real-life data from dust generation monitoring. By pretending and forecasting dust concentrations under differential conditions, the study gives the importance of proactive mitigation measures for dust pollution.
- Asadi et al. (2014) examines the effectiveness of photocatalytic asphalt pavement in removing air pollutants from traffic exhausts. The study employs ANNs and neuro-fuzzy models to analyze the impact of traffic volume, humidity, and solar radiation on predicting NOx concentration in the air. The findings, therefore, are useful in holding the promise toward photocatalytic material in environmental sustainability.
- Jassim et al. (2017) aims to predict hourly energy consumption and CO_2_ emissions of different models of Caterpillar excavators in distinct earthwork conditions using ANNs. The study demonstrates that the ANN model is a suitable technique to forecast energy and CO_2_ consumption and is also capable of predicting energy consumption and greenhouse gas emissions using discrete event simulation data from constructions.
- Hendi et al. (2017) proves the effectiveness of glass powder in stimulating concrete resilience in contrast to sulfuric acid-induced degradation using an ANN model. The study focuses on forecasting the mass and volume reduction of concrete in interaction with acids, thus highlighting the efficiency of glass powder and micro-silica in increasing concrete intensity.
- Kwon and Song (2010) designs a technique to incorporate ANN algorithms with carbonation modeling for deterministic structure behavior. The study focuses on developing a relationship between CO_2_ diffusion rates under the different conditions set for mixture composition and exposure, which leads to the expansion of knowledge on concrete carbonation depth.
- Shahnavaz and Akhavian (2022) develops a ML framework with different algorithms, i.e., ANN, RT, RF and linear regression, to predict emissions by construction equipment from inertial sensors. The objective is to compare the efficiency of different ML models on emission prediction. Accordingly, the RF model is proven to be the most accurate and precise.
- Milivojević et al. (2023) implements IoT technologies for real-time monitoring of air quality at construction sites, focusing on suspended particle concentrations and NO_2_ levels. The study highlights the correlation between construction activities and air pollution, provides insights for mitigating emissions, and demonstrates the feasibility of the IoT-based monitoring system.
- Farahzadi and Kioumarsi (2023) describes the role of AI and ML techniques in predicting CO_2_ emissions in the construction industry, focusing on hyperparameter tuning in estimating them from precast concrete production. The research proves the AI ability to optimize construction operations and decrease environmental impact. The study provides useful insights regarding CO_2_ emission modeling.
- Mäkela et al. (2021) uses wearable sensors to monitor the activity data of the people practicing labor-oriented jobs to enhance construction safety and production. The investigation also sets up ML classifiers that distinguish construction work activities. Thus, these activities aim to achieve practical objectives with the help of safety control based on physiological data.
- Aryal et al. (2017) proposes and develops a wearable sensor-based procedure for real-time monitoring of physical fatigue among construction workers. The physiological data are utilized to analyze the rationale for choosing retrospective sleep EEG indices, estimate the degree of physical fatigue, and show the effectiveness of wearable technologies in modifying work-rest schedules.
- Akhavian and Behzadan (2018) develops a smartphone-based system for monitoring the workers’ movements and activities to overwhelm the problems in input modeling in the simulation models. The study also demonstrates the prospect of applying supervised learning to improve the simulation of worker scenarios by predicting the worker’s actions based on the input sensor data.
- Karataş and Budak (2021) carries out a study to improve construction projects’ general working and management using AI models in activity recognition. Input data are captured from the implemented sensors on employees. Then, data are processed, and ML techniques are used to enhance performance.
- Sherafat et al. (2020) reviews different ML and DL-based systems that can capture construction project activities. This study discusses the use of diverse classifiers to perceive equipment actions and the prospects and issues of automated monitoring systems. Moreover, the paper helps readers understand the current state of activity recognition in the construction industry.
- Akhavian and Behzadan (2016) develops a project management system for construction that incorporates the use of mobile phones’ sensory features to monitor construction workers’ activities. In this paper, the authors use ML to identify construction activities and prove that smartphone sensors could improve work and safety management. According to the findings, sensor-based task recognition is practical.
- Al Jassmi et al. (2020) suggests a wearable technology system to streamline the assessment of the construction worker’s effectiveness. The study focuses on distinguishing between work-related and non-work-related activities using physiological signals, thus underlining the advantages of sensor-based monitoring systems in performance enhancement. This paper also offers important findings on the technologies that can be used to monitor worker’s activities.
- Ouyang et al. (2023) proposes a model that uses the ECG and GSR sensors to predict construction workers’ unawareness resulting from physical fatigue. The paper identifies the physiological measures that can be employed to estimate cognitive functions or tasks. It also indicates how sensor data can be used to track attention levels. Nevertheless, the general assessment indicates that cognitive states are quite effectively predictable in construction.
- Joshua and Koshy (2011) proposes a study that employs accelerometer-based activity classification for automated job sampling on construction sites. The study underlines the necessity of automation to improve construction safety, productivity, and quality control. The study provides significant results. The findings are generalizable beyond masonry tasks. Although the paper discusses masonry, the efficiency of accelerometer-based activity classification may differ amongst building tasks.
- Peddi et al. (2009) creates an automatic productivity measurement system for on-site construction using computer vision and AI principles. The paper demonstrates how AI can speed up construction procedures by automating productivity assessment through real-time image processing.
- Moosavi et al. (2024) conducts a comprehensive survey on the application of Explainable AI in manufacturing and industrial CPS. The paper analyzes the need for transparency and interpretability in AI models used in critical industrial contexts, discussing how Explainable AI techniques enhance the reliability and trustworthiness of intelligent systems in areas like predictive maintenance, cybersecurity, and fault detection. Moreover, the authors categorize Explainable AI methods and assess their role in improving human comprehension and decision-making within industrial environments, suggesting that Explainable AI bridges the gap between advanced AI models and practical deployment in industrial CPS.
- Radanliev et al. (2021) performs a literature review to address the evolving role of AI within CPS and IoT ecosystems, with a particular focus on anomaly detection and system resilience. Additionally, the study proposes a hierarchical conceptual framework for AI decision-making in CPS, suggesting an inevitable evolution of AI cognition due to increased IoT integration. Furthermore, the paper covers challenges in AI transparency and implications for Industry 4.0, arguing for a cascading model that enhances the transparency of AI in Cyber-Physical contexts and outlines AI’s role in safety-critical decision-making for intelligent systems.
- Anumba and Messner (2020) explores the development and implementation of CPS in construction, emphasizing their role in bridging the gap between digital models and physical construction activities. Moreover, the study focuses on utilizing sensors and data acquisition technologies to enable bi-directional coordination, enhancing component tracking, temporary structure monitoring, and mobile crane safety. Hence, the paper presents various prototypes that demonstrate CPS capabilities, highlighting that the construction industry can benefit significantly from these systems through improved real-time feedback and control mechanisms.
- Correa and Maciel (2018) introduces a framework that integrates BIM and CPS for the construction industry, using virtual models based on Petri Nets. Furthermore, the framework connects BIM models to on-site hardware, such as sensors and actuators, to facilitate real-time monitoring and process optimization. It is seen that the paper discusses the application of Industry 4.0 technologies like IoT and big data analytics to construction, aiming to improve automation, enhance monitoring, and simulate processes in a real-time context to reduce manual information gathering and optimize resource allocation on construction sites.
- Anumba et al. (2010) argues for a paradigm shift in the construction industry toward a CPS approach, to improve project delivery through real-time consistency between virtual models and physical construction. Furthermore, the study emphasizes the integration of computational resources like wireless sensors, virtual prototyping, real-time tracking, and data fusion with construction activities. Hence, it proposes a framework for bi-directional coordination, enabling enhanced control and sustainability in construction processes, ultimately reducing inefficiencies and making construction operations more intelligent and streamlined.
- Abdalla and Mohammed (2022) develops predictive models using MARS, MEP, and ANN to estimate the compressive strength of cement-based mortar modified with metakaolin. The MARS model demonstrates superior accuracy, outperforming other techniques. Hence, this model provides a fast, cost-effective prediction of mortar properties, making it suitable for construction material assessments.
- Mohammed et al. (2020) employs ANN, M5P-tree, and NLR to model compressive strength in cement-based mortar containing fly ash. The ANN model shows higher predictive accuracy over NLR and M5P-tree, being effective in enhancing the accuracy of strength prediction in cementitious materials.
- Abdalla and Salih (2022) develops models using MEP, ANN, and M5P-tree to forecast the compressive strength of mortar modified with calcium hydroxide. The MEP model is found to be the most accurate, providing reliable predictions for strength properties, reducing the need for extensive physical testing.
- Mawlood et al. (2021) compares ANN and linear regression models to predict UCS and Cc of clay soils. Both models demonstrate robust predictive abilities for soil properties, with ANN slightly outperforming linear regression. Realizing such AI-based system allows to efficiently evaluate soil strength and reduce dependency on extensive manual testing, thereby expediting geotechnical analyses in construction projects.
- Jaf et al. (2024) examines the effects of waste tire rubber on the compressive strength of concrete through models developed using MEP, ANN, MARS, and NLR. The ANN model outperforms effectively in predicting compressive strength. Hence, the study revealed that increased rubber size significantly affects concrete strength, especially when replacing coarse aggregates.
- Mohammed et al. (2020) utilizes an ANN model to predict the compressive strength of polymer-modified cement-grouted sands. Tests conducted under ASTM and BS standards reveal that the ANN model achieves better results under BS standards. It is noted that the ANN model proves to be more accurate than M5P-tree and other regression-based models for predicting the strength of polymer-modified mixtures.
- Omer et al. (2024) utilizes advanced AI models such as XGBoost, MEP, MARS, and ANN to predict compressive strength in fly ash-modified Recycled Aggregate Concrete (RAC). The XGBoost model exhibits the best performance, whereas sensitivity analysis via SHAP values highlights that curing time, water-to-binder ratio, and cement content are key predictors for RAC strength.
- Wang et al. (2022) combines PCA with an ANN-ABC model to forecast TBM advance rates. The PCA-ANN-ABC model is validated, demonstrating high prediction accuracy. Finally, this model provides a precise method for assessing TBM performance over other regression-based approaches.
- Yu et al. (2021) applies optimized SVM models using WOA and MFO to predict back-break in blasting operations. Moreover, the SVM-MFO model achieves the highest accuracy, outperforming other hybrid SVM models, whereas sensitivity evaluations confirm the SVM-MFO’s effectiveness in forecasting back-break magnitude and enhancing blasting operation safety.
- Jahed Armaghani et al. (2023) explores the performance of different SVM kernel functions in predicting the bearing capacity of deep foundations. It is noted that the study finds that the SVM-RBF kernel achieves the best results. Additionally, the model demonstrates high accuracy and reliability, making it a robust solution for pile bearing capacity prediction in geotechnical engineering.

## 4 Discussion

All the papers reported in Table 1 discuss AI techniques applied to air pollutants and construction materials. Similarly, the papers in Table 2 discuss AI techniques applied to physiological signals in the construction sector. Also, the papers in Tables 3, 4 discuss AI techniques applied, respectively, to CPSs and compressive strength of modeling construction materials.

The interesting observation across the reviewed papers is the presence of various methodologies utilized to determine the construction challenges. Some have used ML and DL algorithms, including RNNs and backpropagation ANNs. In contrast, other studies have used IoT technologies for real-time monitoring or wearable sensors for samples of valid physiological data.

The analyzed research papers discussed and showed the possible applications of AI, ML, and DL techniques in the construction sector. These technologies hold immense potential for addressing challenges in the construction sector, from predicting concentrations of air pollutants and features of construction materials to automating activity recognition and enhancing worker safety. These studies indicate how modern technologies can revolutionize different aspects of construction-related tasks, instilling a sense of optimism for the industry’s future.

The data set collected in each study has been gathered using different setups and sensors. Hence, various experiments collaboratively collected large-scale data on air pollutants, construction materials and physiological signals. The air pollutants data consist of many parameters like demographic and geographic conditions, temperature, pressure, humidity, and gases (e.g., CO_2_, NOx) diffusion coefficients. By contrast, physiological signals data are gathered by measuring heart rate, brain waves, GSR, and standing postures. Finally, the features of construction materials include concrete specimens’ mass loss and volume loss.

Table 1 shows that three papers use DL techniques (Al-Janabi et al., 2019; Mastromatteo and Amelio, 2024; Baduge et al., 2022), which can produce more accurate results than traditional methods. We believe that the reason could be the robustness of this approach, which can better handle noise and outliers in the data. Air pollution is a complex and multifaceted condition with different manifestations and risk factors. AI techniques can handle this complexity by combining various aspects of the data to enable a more comprehensive analysis and enhance the model’s ability to capture the data complexities. The highest-performing methodology is the ANN model by Jassim et al. (2017) with an *R*^2^ value of 0.997 for predicting CO_2_ concentrations. The second one is the backpropagation ANN model by Liu et al. (2022) with an average *R*^2^ value of 0.98 for predicting the dust emission concentration of different work areas of the foundation stage.

Also, Al-Janabi et al. (2019) proposed an effective SAQPM dealing with the data of six pollutants, including PM2.5, PM10, NO_2_, CO, O_3_, and SO_2_. The model has an excellent sMAPE of 0.007 when evaluated over 25 days and thus can be used to enlighten the trends in air quality. Still, Liu et al. (2022) worked on dust emissions at construction sites with high precision but with some drawbacks regarding the scope due to restricted weather conditions and working zones. The model’s specificity highlights a common issue in environmental AI applications: the trade-off between precision and generalizability.

A brief analysis of Table 1 shows that scholars have preferred ensemble methods and models based on ANNs. These models outperform others in the manipulation of huge datasets. Shahnavaz and Akhavian (2022) obtained very high values of *R*^2^ of 0.94 for CO and CO_2_ and 0.91 for NOx using RF, proving that RF, an ensemble method, can handle variability and outliers in data. Likewise, Asadi et al. (2014) used the neuro-fuzzy model compared to ANN to predict NOx concentration; the research found enhanced results of NF model with *R*^2^ measures of 0.97, 0.95, and 0.94 in training, validation, and testing processes, respectively. This indicates that more complicated models are sometimes needed based on the data and the problem at hand. This finding agrees with the view that one must choose the most appropriate model given the characteristics of the data used.

Based on the above, Milivojević et al. (2023) proposed an integration of DL with IoT to predict air quality, which affirms the effectiveness of real-time data in environmental management. However, Jassim et al. (2017) identified this problem in the cross-regional air quality prediction study, and the challenge lies in ensuring the robustness and reliability of these models in different areas and conditions.

Table 1 also reveals that most of the approaches emphasize basic objectives, for instance, managing and monitoring the releases of pollutants with the purpose of determining the levels of CO_2_, NOx, and even H_2_SO_4_. This also contains the method applied by Hendi et al. (2017) and Al-Janabi et al. (2019) where they used ANN and RNN with PSO to investigate the correlation between risk variables and the formation of diverse air pollutants. Finally, Baduge et al. (2022) and Shahnavaz and Akhavian (2022) adopted multi-class classification and regression to evaluate air pollutants.

Shifting the focus to Table 2, the emphasis is on construction worker safety and activity recognition. The innovative use of IoT technology and wearable sensors by Mäkela et al. (2021) achieved impressive classification accuracies (89% for a six-class setup and 78% for a sixteen-class setup). However, the reliance on wearable sensors raises concerns about practicality in real-world construction sites, where maintaining sensor usage consistently can be challenging. For instance, Aryal et al. (2017) employed wearable sensors and boosted tree classifiers to predict physical fatigue, and the accuracy was 82%. Nevertheless, the model might be restricted by the type of sensors used in the dataset.

In the approaches reflected in Table 2, multiclass models prevail to identify the specific states, including fatigue or types of activity. Karataş and Budak (2021) obtained 90% accuracy with the help of an SVM for identifying the worker’s activities, thus proving SVM to be quite useful. However, these models may provide an oversimplified picture of construction sites and their functioning. In their study of inattention assessment in construction workers, Ouyang et al. (2023) employed ECG and GSR sensors, thus increasing the accuracy to 96.67% with the SVM algorithm demonstrating the efficacy of using multiple features.

ML methods discussed in the paper by Akhavian and Behzadan (2018) for workers’ activity recognition combine sensor data, activity logs, and environmental factors, allowing for detailed analysis of construction site dynamics. By contrast, Akhavian and Behzadan (2016) automated construction task identification using smartphone sensors, achieving over 90% accuracy across various activities. This demonstrates AI’s potential to boost productivity and safety on construction sites. However, reliance on smartphone sensors might limit applicability, as workers cannot always carry or interact with these devices.

Regarding data, it is worth noting that most techniques are based on the worker’s activity. Among these techniques, three out of four are based on ANN (Peddi et al., 2009; Al Jassmi et al., 2020; Akhavian and Behzadan, 2018), DT (Joshua and Koshy, 2011), and vision-based methods (Sherafat et al., 2020). These techniques are adopted for activity identification and co-occurrence, while two of them (Aryal et al., 2017; Ouyang et al., 2023) aim to establish methods for fatigue.

From a comparison of Tables 1, 2, we can observe that most of the approaches in Table 1 are related to predicting air pollutants using regression strategies. Only one paper in Table 1 (Hendi et al., 2017) aims to predict the concrete specimens’ mass loss and volume loss in the construction site. By contrast, the approaches based on physiological activity in Table 2 are mainly related to the identification of worker’s gestures (see Aryal et al., 2017; Al Jassmi et al., 2020; Akhavian and Behzadan, 2018; Karataş and Budak, 2021; Joshua and Koshy, 2011; Peddi et al., 2009).

Regarding the complexities of the analysis of air pollutants, we can observe that Asadi et al. (2014) and Jassim et al. (2017) used input data characterized by a specific duration of time. It is worth noting that both Liu et al. (2022) and Hendi et al. (2017) adopted a neural learning model (i.e., backpropagation), which is a more robust and reliable approach than traditional classifiers. Baduge et al. (2022) used GANs and VAEs, which are DL methods specifically adopted for prediction tasks on temporal data. In the determination of real-time monitoring of the worker’s performed tasks on the construction site by physiological signal processing, Sherafat et al. (2020) described the use of CNN with a vision-based method model, a highly potent and effective network for a real-time demonstration of the performed task of the workers. Likewise, Akhavian and Behzadan (2018) analyzed and classified the data gathered from the cell phone’s gyroscope and accelerometer motion sensor. Finally, Al Jassmi et al. (2020) presented an additional novel solution for how remote-generated physiological signals and activity can be automatically provided.

Table 3 showcases the transformative role of AI and CPS in enhancing cybersecurity and operational efficiency in the construction industry.

In particular, Anumba et al. (2010) emphasized the integration of CPS with virtual models for real-time accuracy on construction sites, reducing costly errors and improving project outcomes. Similarly, Anumba and Messner (2020) advocated for a comprehensive integration of information technologies into project delivery, promoting sustainability and intelligence in construction processes.

Still, Moosavi et al. (2024) highlighted the need for Explainable AI in construction, ensuring transparency and trust in AI decision-making. On the other hand, Correa and Maciel (2018) discussed the optimization of planning and real-time monitoring through BIM, while Radanliev et al. (2021) presented a framework for AI decision-making that enhances resilience against cyber threats.

Finally, the diverse methodologies across these studies, from real-time monitoring with sensors to systematic classifications of Explainable AI techniques, reflect the multifaceted challenges faced in the construction sector.

Recent research has significantly advanced the prediction of compressive strength in various construction materials, reflecting a trend toward improved modeling techniques and sustainability in construction practices as comprised in Table 4.

Abdalla and Mohammed (2022) began by exploring the effects of metakaolin on mortar strength through the analysis of 230 data samples. Their findings revealed that MARS model outperformed others in accuracy, highlighting the importance of model selection in predicting material properties.

In contrast, with the perspective of material modification, Mohammed et al. (2020) focused on fly ash-modified mortar, analyzing 450 samples and underscoring the critical role of curing time in strength development. They found that NLR and ANNs provided the most accurate predictions, illustrating how advanced ML techniques enhance predictive capabilities in concrete research.

In a complementary study, Abdalla and Salih (2022) investigated calcium hydroxide in mortar, demonstrating that while calcium hydroxide can improve certain properties, excessive amounts may reduce compressive strength. Hence, this finding emphasizes the need for careful material composition, giving balance between different additives and the resulting material performance.

Shifting focus to soil materials, Mawlood et al. (2021) developed predictive models for clay soils and discovered that linear regression models could achieve performance comparable to ANNs. Their research identified dry density as a key factor, suggesting that simpler models can be effective in specific contexts, thus offering accessible solutions for practitioners in geotechnical engineering.

In comparison, Jaf et al. (2024) further expanded the discussion on sustainability by addressing the incorporation of recycled tire rubber in concrete. Although their findings indicated that rubber could reduce compressive strength, the ANN model effectively predicted the strength of rubberized concrete, thus promoting recycling practices in construction and showcasing innovative uses for waste materials.

Similarly, Mohammed et al. (2020) examined how different sand grain sizes impact the compressive strength of polymer-modified cement grouts. Moreover, they found that coarser sands yield higher strengths at lower water/cement ratios, reinforcing the idea that aggregate properties significantly influence strength predictions, echoing earlier findings on the importance of material characteristics.

In the pursuit of eco-friendliness, Omer et al. (2024) investigated the use of fly ash and recycled aggregates in concrete. Furthermore, their study concluded that the XGBoost model provided the best predictions, having *R*^2^ of 0.97, with curing time identified as a crucial factor, thus aligning with previous findings on the importance of curing conditions in achieving optimal strength.

Moreover, Wang et al. (2022) contributed to the field by utilizing PCA to enhance predictions for TBM performance. Additionally, their PCA-ANN-ABC model achieved good accuracy with an *R*^2^ of 0.96, demonstrating the effectiveness of integrating statistical methods with ML to address engineering challenges, paralleling the advancements made in other studies.

Considering the theme of optimization, Yu et al. (2021) developed optimized SVM models to forecast back-break caused by blasting. They proved that SVM-MFO model outperforms other methods, with an *R*^2^ of 0.95 and RSME of 0.45, illustrating how advanced algorithms can tackle specific issues in mining and construction.

Finally, Jahed Armaghani et al. (2023) focused on predicting pile bearing capacity and found that the SVM-RBF model provided the highest reliability in predictions with *R*^2^ of 0.99 on the test set. Hence, this study underscores the critical role of robust modeling techniques in ensuring safety and efficiency in construction projects and tying back to the overarching theme of improving prediction accuracy across various construction materials.

In a nutshell, the models presented in Table 1 intend to predict the environmental conditions and emissions with considerable reliability, whereas Table 2, on the other hand, relies on wearable and sensor technologies to improve the safety of the construction workers and their activity identification. Similarly, the papers in Table 3 have a significant importance in the construction industry, as AI and CPS technologies ultimately foster a more integrated approach to project management and delivery. Finally, papers comprised in Table 4 reflect a comprehensive effort to enhance predictive modeling in construction materials, and careful material selection to drive innovation in the industry. This indicates that applying AI and ML techniques encompasses almost all industries. The research studies underscore critical scalability, practicality, and generalizability challenges that need addressing to realize these technologies’ potential in real-world scenarios. Moreover, these approaches promote the safety enhancement, efficiency, and resilience, emphasizing the integration of advanced methodologies, and sustainable practices.

## 5 Conclusion

**Figure 3:**
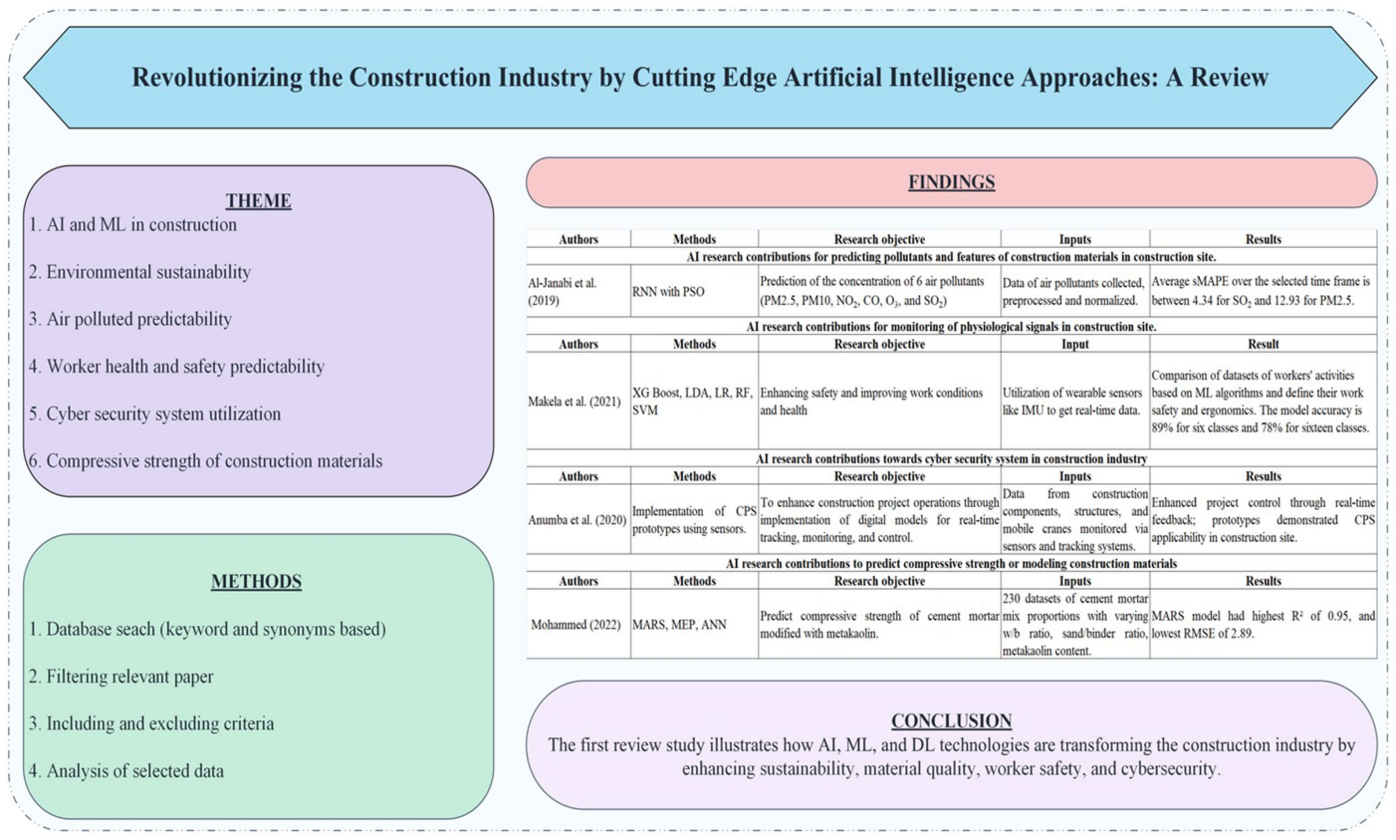
Detailed graphical abstract of the proposed review.

This review study brings together a hub of research on how AI and ML are being used in the construction industry, showcasing their powerful impact on key areas like sustainability, CPS implementation, material quality, and worker safety. Additionally, it provides a clear understanding of where the field stands now and where it might be headed. To make this analysis easy to follow, the conclusion is divided into five parts: findings, recommendations, and limitations, implementation of the current study and summary of novelty and contribution offering a structured look at the present state and future possibilities of AI in construction. A detailed graphical abstract of the findings and conclusion of the present study is shown in Figure 3.

### 5.1 Findings

This review highlights how AI and ML technologies are reshaping the construction industry by offering groundbreaking solutions in four main areas: sustainability, material quality, worker safety, and CPS. AI models, such as ANN and SVM, have shown remarkable accuracy in predicting complex variables like air pollutant levels (*R*^2^ = 0.997 for CO_2_ emissions) and material strength. These models demonstrate their ability to handle intricate data and address real-world challenges.

Beyond predictive analytics, AI has a crucial role in materials science. It optimizes the use of recycled materials like fly ash and rubber, balancing sustainability with structural integrity. For worker safety, advanced models using IoT, and wearable sensors allow real-time monitoring of physiological signals such as heart rate and brain activity, enabling timely interventions to reduce risks like fatigue. In CPS, AI-driven systems have greatly improved threat detection and response, ensuring that construction projects are protected from emerging digital threats. By integrating these technologies, the construction sector can significantly enhance efficiency, reduce its environmental footprint, improve material quality, and protect workers, marking a new era in smarter, more resilient construction practices.

### 5.2 Recommendations

Besides, regarding the current data on air pollutants, most strategies in Table 1 involve a linear or non-linear regression problem to identify pollutant levels. We believe that regression analyses and more advanced multi-class approaches could bring more insightful results. In conclusion, the approaches in Table 2, aiming to detect physiological signals and worker activities, are quite different from those in Table 1, focusing on air pollutants, environmental factors and construction materials. Also, note that most works in Table 1 deal with predicting environmental factors and emissions and identifying the concentration of pollutants like CO_2_, NOx, and H_2_SO_4_. We believe that predicting prognosis in construction worker safety and activity recognition could be interesting for understanding the duration and severity of physiological responses. For example, DL analyses could detect early signs of worker fatigue or stress using temporal data such as heart rate and brain wave monitoring over time, allowing timely interventions and personalized safety measures to improve worker outcomes.

### 5.3 Limitations

Despite the potential, the AI models discussed in this review face some limitations. Many rely on small, localized datasets, which restrict their use in different geographic and climatic conditions. Models like the one by Liu et al. (2022), tailored to specific environments, may struggle to generalize across broader contexts. The reliance on historical data makes it hard to predict future unknown variables, and the complexity of AI models can limit their practical use in the fast-changing construction environment. In terms of CPSs, Anumba and Messner (2020), some models may lack the flexibility to tackle evolving threats. Similarly, AI models for material performance Radanliev et al. (2021) often do not consider long-term durability under changing conditions. While models like Ouyang et al. (2023) show promise for tracking worker fatigue, scaling them to real-world construction sites, where continuous sensor use may not be feasible, presents a challenge.

### 5.4 Implications of the current study

This review underscores AI’s game-changing role in solving critical construction challenges. From improving material quality and reducing environmental pollutants to enhancing worker safety and boosting cybersecurity, AI offers powerful tools that can make construction projects more sustainable, efficient, and secure. However, to fully harness these technologies in large-scale, real-world scenarios, it is essential to address current limitations in scalability, data availability, and adaptability.

### 5.5 Summary of novelty and contribution

This is the first comprehensive review that explores the use of AI, ML, and DL in areas such as air quality management, material performance, worker safety, and CPS within the construction industry. It identifies significant research gaps—such as model scalability and reliance on specific datasets—and provides a roadmap for future studies. By showcasing the diverse applications of AI and its potential to revolutionize construction practices, this review highlights both the immense opportunities and challenges in integrating AI across the sector.
